# Sedentariness and Health: Is Sedentary Behavior More Than Just Physical Inactivity?

**DOI:** 10.3389/fpubh.2018.00258

**Published:** 2018-09-10

**Authors:** Shirin Panahi, Angelo Tremblay

**Affiliations:** ^1^Department of Kinesiology, Université Laval, Québec City, QC, Canada; ^2^Department of Physical Education, Université Laval, Québec City, QC, Canada; ^3^Centre de Recherche de l‘Institut de Cardiologie et de Pneumologie de Québec, Québec City, QC, Canada

**Keywords:** sedentary behavior, mental work, diet, physical inactivity, sit-stand desks, exercise pause, physical activity participation

## Abstract

Sedentary behavior refers to certain activities in a reclining, seated, or lying position requiring very low energy expenditure. It has been suggested to be distinct from physical inactivity and an independent predictor of metabolic risk even if an individual meets current physical activity guidelines. Over the past decades, a shift in the activity profile of individuals has been observed with vigorous physical activity and sleep being partly replaced by cognitive work, a potential neurogenic stress component considering its hormonal and neurophysiological effects, leading to various impacts on health. Mental work, for instance, may significantly increase glycemic instability leading to an increase in the desire to eat and thus, higher energy intakes. Furthermore, screen-based leisure activities (e.g., television watching) and screen-based work activities (e.g., computer use for work purposes) have often been considered together while they may not trigger the same stress response and/or use of substrate. Thus, the problems of sedentariness may not only be attributed to a lack of movement, but also to the stimulation provided by replacing activities. The objective of this review is to discuss the (1) recent evidence and current state of knowledge regarding the health impact of sedentary behaviors on health; (2) potential neurogenic effects of cognitive work as a sedentary behavior; (3) link between sedentary behaviors and the diet; (4) resemblance between sedentary behaviors and the inadequate sleeper; and (5) potential solutions to reduce sedentary behaviors and increase physical activity.

## Introduction

Although the beneficial health effects of physical activity have been well recognized, physical inactivity accounts for 9% of premature mortality worldwide (1). The term physical inactivity refers to performing insufficient amounts of moderate to vigorous-intensity activity (i.e., not meeting specific physical activity guidelines) (2). Sedentary behavior, on the other hand, has been suggested to be distinct from physical inactivity and an independent predictor of metabolic risk even if an individual meets current physical activity guidelines (3). The World Health Organization recommends that adults aged 18 or older participate in at least 150 min of moderate-to-vigorous activity per week or the equivalent of 30 min of daily activity (4). Currently, just over 15% of Canadian adults are meeting these guidelines (5). However, it is unclear if meeting these guidelines of activity is sufficient to be considered non-sedentary. Daily physical activity levels are evaluated by a person's daily energy expenditure divided by his or her basal metabolic rate (6). The prevalence of sedentary behavior, defined as any waking behavior that requires low energy expenditure (≤1.5 MET) such as prolonged sitting, reclining or lying down (2), is very high in developed countries. Results from the 2003/2004 National Health and Nutrition Examination Survey (NHANES) demonstrated that children and adults in the United States spend ~7.7 h/day of their waking time engaged in sedentary behaviors such as watching television, playing passive video games, using the computer, prolonged sitting (e.g., at a desk) and motorized transportation (7).

Over the last 30 years, overweight and obesity have become characteristic of the majority of Canadians which has led to a concomitant increase in the prevalence of co-morbidities including type 2 diabetes and cardiovascular disease. Sedentary behavior has been a contributing factor to this epidemic and associated with an increased risk of all-cause mortality (8, 9). In a 12-year prospective study, a progressively higher risk of mortality was found across higher levels of sitting time from all causes and cardiovascular disease, independent of leisure-time physical activity (10). Furthermore, in a meta-analysis of six studies evaluating daily sitting time and all-cause mortality, a 34% higher mortality risk for adults sitting 10 h/day was observed after taking physical activity into account (9). Sedentary behavior has also been linked with poor glycemic control including a reduction in insulin sensitivity and glucose uptake (11). Several animal studies have shown that insulin-mediated glucose uptake is significantly reduced due to muscular inactivity (12, 13). Epidemiological studies have consistently reported that time spent in sedentary tasks that require little muscular activity (low accelerometry counts, computer use or self-reported television time) is negatively associated with insulin action (14, 15). In a clinical trial, healthy non-exercising young men who reduced their daily activity levels from normal (10,501 steps/day) to low (1,344 steps/day) levels of ambulatory activity for 2 weeks were found to display metabolic alterations including a 17% decline in their insulin sensitivity (16). However, because higher inactivity decreases energy expenditure, if this reduction is not compensated for by a reduction in energy intake it will lead to energy surplus which has been shown to increase insulin resistance (17).

In addition to the changes in human activity, globalization and technological changes have favored a progressive switch from physically demanding tasks to knowledge-based work or mental activity soliciting an enhanced cognitive demand. Screen-based leisure activities (e.g., television watching, video games, and internet use) and screen-based work activities (e.g., computer use for work purposes) have often been considered together while they may not trigger the same stress response and/or use of substrate. Furthermore, from a physiological perspective, the biological requirements and effects of physical and cognitive work are not the same. Mental work, for instance, may significantly increase glycemic instability (i.e., wide fluctuations in blood glucose concentrations) leading to an increase in the desire to eat and thus, higher energy intakes (18, 19). Thus, the problems of sedentariness may not only be attributed to a lack of movement, but also to the stimulation provided by replacing activities. In a context where there is exposure to cognitive work, novel strategies to increase physical activity and improve energy balance regulation are needed.

Therefore, the objective of this review is to discuss the (1) recent evidence and current state of knowledge regarding the health impact of sedentary behaviors on health; (2) potential neurogenic effects of cognitive work as a sedentary behavior; (3) link between sedentary behaviors and the diet; (4) resemblance between sedentary behaviors and the inadequate sleeper; and (5) potential solutions to reduce sedentary behaviors and increase physical activity.

## Epidemiological observations

The energy cost of various activities, both in work and leisure, has been of great interest to researchers. Regular physical activity has been associated with decreased adiposity (20, 21), an increase in muscle oxidative potential (22) and resting metabolism (21), a decrease in energy intake relative to energy expenditure (23) and an increase in beta-adrenergic stimulation (21, 24). In a study examining the association between physical activity and weight loss maintenance in a group of individuals who were previously living with obesity, participants in the highest tertile of physical activity (highly active; >1575 kcal/week) experienced a significantly lower weight regain compared to those in the low (< 850 kcal/week) and moderately (850–1575 kcal/week) active groups after a two year follow-up (25). This suggests that significant weight loss may be maintained for 2 years when weekly caloric expenditure is greater than 1500 kcal/week; however, success also depending upon the degree of lifestyle changes made. This study also indicated that the increasing the frequency of exercise appeared to be the best method for increasing weekly caloric expenditure and that increased fat utilization post-exercise may be a likely contributor to maintaining a lower body weight long term. Inverse associations have been observed between time spent in moderate-to-vigorous physical activity and indices of adiposity in children, independent of objectively measured sedentary time and other covariates, while sedentary behavior was not linked with any of the adiposity indicators (26). However, frequent interruptions in sedentary time have been shown to be associated with a favorable cardiometabolic risk profile in adults (27) and among children with parental obesity (28). In a cross-sectional study of children with a family history of obesity, examining the associations among moderate-to-vigorous physical activity, fitness, sedentary behavior and insulin sensitivity using two markers of characterization (i.e., accelerometer and screen-time), it was found that physical activity was correlated with indices of insulin sensitivity independent of fitness and sedentary behaviors; however, this association was attenuated when adiposity was considered (29). Furthermore, self-reported screen-time was negatively associated with insulin sensitivity in girls, but not boys, after controlling for physical activity, fitness and adiposity (29). Although the reason for this is not clear, other factors including dietary habits linked to screen-time were suggested to be involved which may explain its effects on insulin sensitivity (29). Additionally, as previously discussed, this suggests that a stress-related biological reality related to screen-time may also promote metabolic dysfunctionality. In a recent meta-analysis of 16 studies, high levels of moderate intensity physical activity (60–75 min/day) appeared to offset the increased risk of mortality associated with high sitting time; however, the high activity did not eliminate the increased risk associated with television viewing suggesting the importance of considering the type of activities while sitting (30).

Extended periods of sedentary behavior results in low energy expenditure and may contribute to weight gain and negative health effects via effects on energy intake. Changes in energy expenditure and energy intake have been attributed to many factors including changes in family dynamics and popular sedentary activities including using computers and television viewing. In a systematic review of observational studies, higher levels of sedentary behavior (e.g., television viewing) were associated with a less healthful diet, such as less fruit and vegetable intake and higher consumption of energy-dense snacks and sugar-sweetened beverages in pre-school and school-aged children and adolescents (31). However, the results were less conclusive in adults. Technological development has favored a progressive switch from physically demanding tasks to knowledge-based work, soliciting great cognitive demand (32). This may be reflected by activities such as computer “chatting” in children, whereas for adults, it may represent knowledge-based work that appears to be essential from the perspective of economic competitiveness (i.e., labor efficiency and productivity) (33).

## Effects of sedentary behavior vs. physical inactivity on energy intake, appetite control and metabolism

Over time, a shift in the activity profile of individuals has been observed. Vigorous physical activity and sleep have been, in part, replaced by cognitive work which has also contributed to various health-related effects. Sedentary occupations have become the norm with approximately one in two individuals performing primarily sedentary tasks. In addition to the low energy expenditure from these sedentary tasks, high mental demands at work have been associated with increased food intake suggesting that this may lead to a positive energy balance (34–36). In a study examining the impact of knowledge-based work on spontaneous energy intake, subjective appetite and glucose homeostasis, healthy women students were randomly assigned to one of three 45-min conditions including (1) resting in a seated position; (2) reading a document and writing a summary; or (3) performing a battery of computerized tests followed by an *ad libitum* buffet meal (18). Although no differences in subjective appetite were observed, mean energy intake following the reading-writing and automated test-battery conditions exceeded that measured after rest by 203 kcal and 253 kcal, respectively (18). Furthermore, significant variations in plasma glucose and insulin concentrations were observed compared to the seated only position suggesting that this may be considered a risk factor for a positive energy balance leading to overweight in the longer term (18). Cortisol concentrations over the 45 min in the two cognitive conditions was also significantly higher compared to the control condition suggesting knowledge-based work as a neurogenic stress component considering its hormonal and neurophysiological effects. Activation of the hypothalamic-pituitary-adrenal (HPA) axis is the primary neuroendocrine response to both psychological and physiological stress and previous studies (37, 38) have shown that stress-induced cortisol reactivity is associated with greater food intake which may explain the response to the knowledge-based conditions in this study (18). In another study assessing the impact of a major work deadline (high workload) and a quiescent period of work (low workload) on plasma lipids, dietary intake, and self-reported stress in employees, self-reported stress, plasma total cholesterol, energy and dietary fat intakes were higher in the high workload compared to the low workload condition (34). Although the association between cognitive work and body weight has been primarily investigated in adults, one study examining the link between homework duration, adiposity indicators, and stress-related levels in school-aged children found that boys with a high workload of homework, when combined with schoolwork-related stress, had unfavorable adiposity indicators (i.e., higher percent body fat) (39). As has been previously suggested, from a physiological perspective, the biological requirements of physical and mental work are different because knowledge-based work is a type of activity that relies on the brain which utilizes glucose for the metabolism of energy compared to physical activity which uses skeletal muscle and relies mostly on fat metabolism, depending on the type of physical activity (40). For example, frequent interruptions of prolonged sitting with short bouts of activity rely primarily on carbohydrate as fuel. However, in a study by Volkow et al., positron emission tomography was used to examine the impact of methylphenidate medication on the amount of glucose required by the brain to perform a cognitive task (41). It was found that methylphenidate reduced the increase in carbohydrate utilization induced by mental work by ~50% (41).

Appetite control occurs through a complex interaction between physiology and behavior. Low physical activity levels have been suggested to interact with body fat to dysregulate appetite and be a source of overconsumption (42, 43). Hormonal responses to changes in energy intake and structured exercise have been observed; however, few studies have investigated their responses to increased time spent in sedentary activities. In a clinical trial of non-obese adults, only one day of inactivity, long hours of sitting, and minimal walking or standing, decreased insulin sensitivity even when energy intake was reduced to maintain energy balance (44). Subjects participated in three study sessions, mostly sitting without matching energy intake (SIT), sitting with matching energy intake (SITBAL), and no sitting (NO-SIT) (45). Three meals, breakfast, lunch, and dinner were exactly the same between SIT and NO-SIT. However, the caloric content of the breakfast and lunch were reduced by about 1,000 calories to match the reduction in energy expenditure in SIT-BAL. The next morning insulin action was tested. The results indicate that whole body insulin action was lower in SIT and SIT-BAL compared to NO-SIT (39 and 18% respectively). Therefore, both muscle inactivity and energy surplus contribute to the effect of prolonged sitting on insulin action. Further analyses examining gastrointestinal hormone response showed that SIT-BAL led to an increase in ghrelin in the men, but attenuated the leptin response, reduced ghrelin, increased hunger, and decreased fullness in the women. Because a reduction in energy expenditure was not accompanied by lower appetite, prolonged sitting may promote excess energy intake, leading to weight gain in both men and women. Physical inactivity has also been shown to interact with dietary macronutrient composition to influence energy and fat balance (46). Energy intake was not found to be regulated over a 2-day period in response to either imposition of inactivity or a high-fat diet (46). It was suggested that physical activity was essential to the avoidance of a significant positive energy balance.

**Figure 1:**
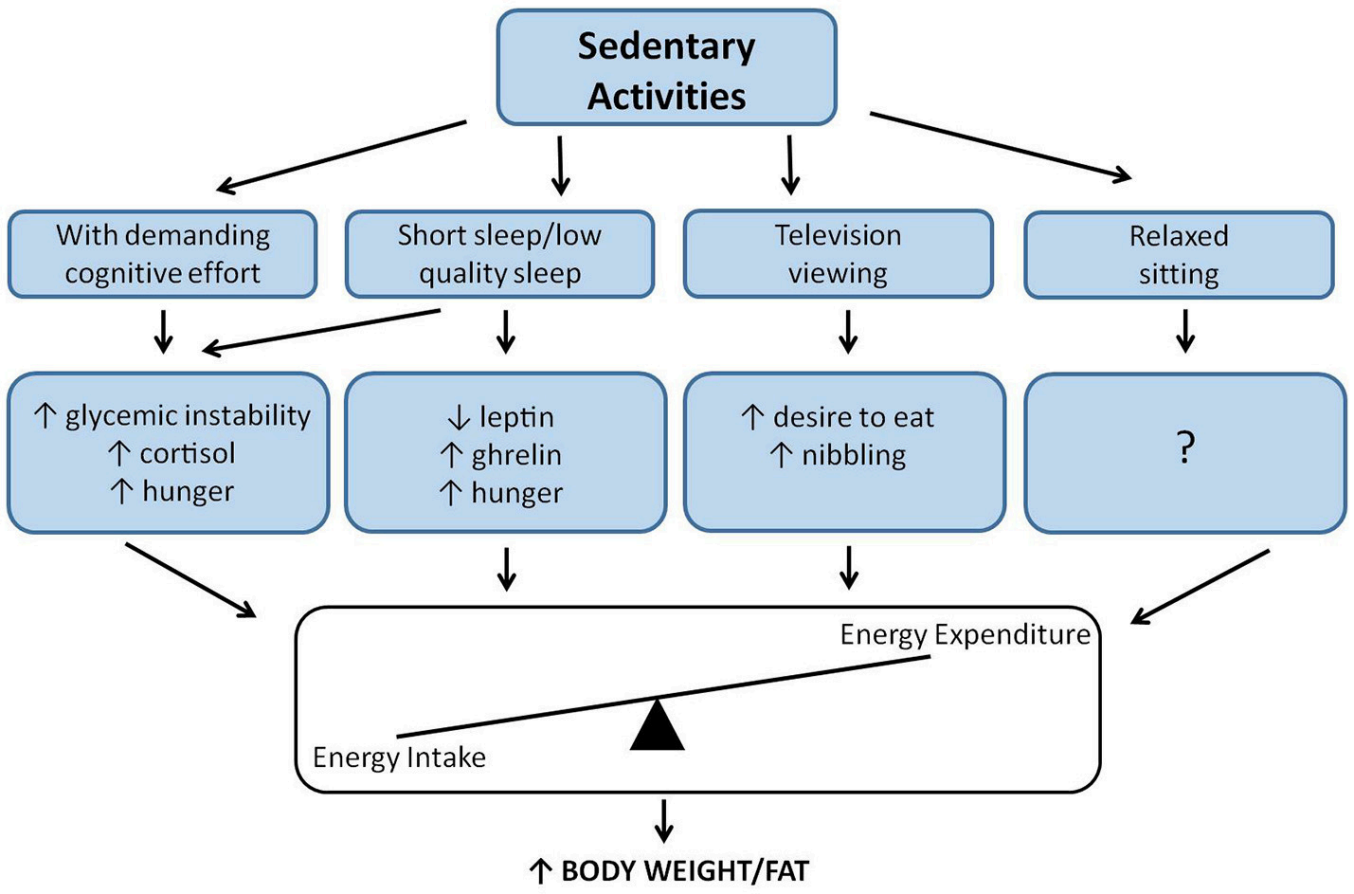
Sedentary behavior and effects on appetite and glycemic control.

From a practical standpoint, sedentary behavior is frequently associated with activities performed in a seated position. As previously discussed, there are potentially unfavorable stimulations that may be promoted by seated activities. However, these observations also reveal that the main problem of sedentariness in this context is maybe not the seated position, but rather the stressful stimulation that would accompany seated activities. For instance, in the context of usual daily activities seated labor can become stressful because of demanding cognitive effort, an inadequate sitting position, a stressful labor environment, or seated work that may be too long. Up until now, the biological mechanisms underlying stressful sitting activities have not been sufficiently documented; however, as discussed, our research experience suggests that stressful sitting may promote glycemic instability, hypercortisolemia and a reduced parasympathetic activity (18), which are all biological adaptations that are contrary to optimal metabolic fitness and body composition. Furthermore, up until now, there is no clear evidence that reading an interesting book in a seated position to relax before going to bed has negative effects (Figure 1).

## Sedentary behavior and the inadequate sleeper

There is a resemblance between the biological effects of inadequate sleep compared to stressful seated work. In individuals with inadequate sleep habits, for example, glycemic instability has also been documented (47) and it is also well-known that short sleepers (< 6 h/night) are more prone to excessive energy intake and thus, weight gain compared to individuals sleep 7–8 h/night (48). These observations add to the proof of concept, where it is not so much the nature of the sedentary activity, but the stress-related biological reality that may be related to it.

## Potential solutions to counteract the apparent detrimental effects of sedentariness

Clinical and public health guidelines for physical activity have been in place for nearly two decades (49); however, no quantitative guidelines exist for sedentary behavior because it is not known how much sedentary behavior is harmful to health. The impact of cognitive work as a sedentary behavior appears to be a stimulus favoring a significant enhancing effect on food intake and very trivial effects on energy expenditure. There is a clear disturbance in the context of modernity because of what we are accustomed to due to evolution. We have been configured to be hunter-gatherers and now we have chosen a modality of labor that is not optimally adapted to what we are used to. It would be difficult to return to the way of living of our grandparents using the “technology” of the past; however potential solutions that consider approaches to counteracting the negative impact of mental work may be possible with the readjustment of daily physical activity schedules.

In the context of a school or work environment, recent data has suggested that combining mental and physical work (e.g., active pauses/meetings), may be one strategy to reduce sedentary time in a context where potential neurogenic stress may be high. Both mental work and physical activity can influence hunger and food intake by producing various physiological changes. For example, an active exercise pause between a session of mental work and a buffet meal on energy intake and energy balance was found to represent a strategy to create a negative energy balance via an increase in energy expenditure and maintenance of energy intake (50). Furthermore, an acute bout of interval exercise after mental work was shown to decrease food consumption compared with a non-exercise condition suggesting that it may be used as an approach to offset positive energy balance induced by mental tasks (51). The school environment represents a good place for children and adolescents to improve the balance between physical activity and the cognitive demands of mental work. Students registered in a traditional sports studies program in ice hockey (school-related sessions in the morning and hockey-related activities in the afternoon) were found to experience a decrease in their body mass indices and increase in their aerobic and muscular fitness over the academic year without compromising their academic success (52). A physical education class prior to mental work was found to reduce blood pressure suggesting that physical education should be more prominent in schools and part of children‘s daily activities (53). Furthermore, elementary school children were found to be more physically active in an activity-permissive school environment up to the conditions of summer vacation conditions compared with a traditional school with chairs and desks and traditional school with desks which encouraged standing (54). The possibility of moving and even standing are good examples of modalities of cognitive work in which there is an inclusion of movement. The same scenario could be considered in a professional environment where active meetings could be developed to permit individuals to talk while walking or cycling on relevant machines.

Several years ago, our research group began active research meetings and examined its impact on perceived stress in staff and students at Laval University in Quebec City. A beneficial effect on self-reported stress and performance was found among staff and student members of the group suggesting that this may be one way to increase physical activity participation and improve the unfavorable effects of sedentariness on overall health. Computer-related activities which are common in both the school and work environment, for example, represent particular types of sedentary activities that are stressful and biologically demanding and thus, “re-designing” these environments may be essential to promoting more movement (54, 55).

Because sitting is widespread among desk-based activities, ergonomic adaptations including sitting-standing desks while working may be an approach that may decrease the negative effects of sedentary activities. Although the effects of sitting and standing on metabolism (e.g., blood pressure, glucose, and lipid metabolism) and cardiovascular risk is almost the same, evidence suggests that breaking up sedentary time with standing may be sufficient to improve productivity, relieve lower back pain and increase movement (56). In the workplace, sit-stand desks were found to be effective in decreasing workplace sedentary behavior in office workers with abdominal obesity, with no change in sedentary behavior or physical activity outside of work hours; however, these changes did not alter markers of cardiometabolic risk in these individuals (57). Furthermore, the use of sit-stand desks in sedentary office workers was also associated an overall sense of well-being and energy, decreased fatigue, and reduction in appetite, food intake and lower self-perceived levels of hunger (58). Introducing sit-stand desks was also shown to increase classroom standing time among university students who reported improvements in engagement, participation, attention and declines in restlessness, fatigue, boredom and cell phone use (59).

## Conclusion

Based on the available evidence, sedentary behavior may be more than just physical inactivity. Calorie for calorie we deal with a profile of stimulation that may not necessarily have the same effects on appetite control, related peripheral biomarkers, and neuro-messengers. There appears to be a modern version of sedentary behavior that bears a potential neurogenic component leading to hyperphagia, stress, and unfavorable metabolic health outcomes; however, various approaches may help to increase physical activity participation that may possibly counteract the apparent unfavorable effects of sedentary behaviors. In the context of an environment where we are submitted to desk-based and computer-related activities, we must preserve our movement for optimal health and implement some of these strategies to increase physical activity participation in our schools and workplaces. Thus, socio-ecological interventions that consider multiple components are needed to help reduce sedentary behaviors and promote physical activity.

## Author contributions

AT was responsible for the content of the manuscript. SP and AT wrote, read and approved the final manuscript.

### Conflict of interest statement

The authors declare that the research was conducted in the absence of any commercial or financial relationships that could be construed as a potential conflict of interest.

## References

[1] Lee IM Shiroma EJ Lobelo F Puska P Blair SN Katzmarzyk PT. Effect of physical inactivity on major non-communicable diseases worldwide: an analysis of burden of disease and life expectancy. Lancet (2012) 380:219–29. 10.1016/S0140-6736(12)61031-9 22818936 PMC3645500

[2] Tremblay MS Aubert S Barnes JD Saunders TJ Carson V Latimer-Cheung AE. Sedentary behavior research network (SBRN) - terminology consensus project process and outcome. Int J Behav Nutr Phys Act. (2017) 14:75. 10.1186/s12966-017-0525-8 28599680 PMC5466781

[3] Booth F Lees S. Fundamental questions about genes, inactivity, and chronic diseases. Physiol Genomics (2007) 28:146–57. 10.1152/physiolgenomics.00174.2006 17032813

[4] World Health Organization Global Recommendations on Physical Activity for Health. Geneva (2012). 26180873

[5] Statistics Canada Physical activity levels of Canadian adults, 2007 to 2009. (2009) Available online at: http://www.statcan.gc.ca/pub/82-625-x/2011001/article/11552-eng.htm.

[6] Food and Agriculture Organization of the United Nations Human Energy Requirements: Principles and Definitions. Rome (2004).

[7] Matthews CE Chen KY Freedson PS Buchowski MS Beech BM Pate RR. Amount of time spent in sedentary behaviors in the United States, 2003-2004. Am J Epidemiol. (2008) 167:875–81. 10.1093/aje/kwm390 18303006 PMC3527832

[8] Bjork Petersen C Bauman A Gronbaek M Wulff Helge J Thygesen LC Tolstrup JS Total sitting time and risk of myocardial infarction, coronary heart disease and all-cause mortality in a prospective cohort of Danish adults. Int J Behav Nutr Phys Act. (2014) 11:13 10.1186/1479-5868-11-13 24498933 PMC3922425

[9] Chau JY Grunseit AC Chey T Stamatakis E Brown WJ Matthews CE. Daily sitting time and all-cause mortality: a meta-analysis. PLoS ONE (2013) 8:e80000. 10.1371/journal.pone.0080000 24236168 PMC3827429

[10] Katzmarzyk PT Church TS Craig CL Bouchard C. Sitting time and mortality from all causes, cardiovascular disease, and cancer. Med Sci Sports Exerc. (2009) 41:998–1005. 10.1249/MSS.0b013e3181930355 19346988

[11] Diaz KM Goldsmith J Greenlee H Strizich G Qi Q Mossavar-Rahmani Y. Prolonged, uninterrupted sedentary behavior and glycemic biomarkers among US hispanic/latino adults: the HCHS/SOL (Hispanic Community Health Study/Study of Latinos). Circulation (2017) 136:1362–73. 10.1161/CIRCULATIONAHA.116.026858 28835368 PMC5634934

[12] Seider MJ Nicholson WF Booth FW. Insulin resistance for glucose metabolism in disused soleus muscle of mice. Am J Physiol. (1982) 242:E12–8. 10.1152/ajpendo.1982.242.1.E12 7058883

[13] Ploug T Ohkuwa T Handberg A Vissing J Galbo H. Effect of immobilization on glucose transport and glucose transporter expression in rat skeletal muscle. Am J Physiol. (1995) 268(5 Pt 1):E980–6. 10.1152/ajpendo.1995.268.5.E980 7762654

[14] Healy GN Dunstan DW Salmon J Shaw JE Zimmet PZ Owen N. Television time and continuous metabolic risk in physically active adults. Med Sci Sports Exerc. (2008) 40:639–45. 10.1249/MSS.0b013e3181607421 18317383

[15] Healy GN Dunstan DW Salmon J Cerin E Shaw JE Zimmet PZ. Objectively measured light-intensity physical activity is independently associated with 2-h plasma glucose. Diabetes Care (2007) 30:1384–9. 10.2337/dc07-0114 17473059

[16] Krogh-Madsen R Thyfault JP Broholm C Mortensen OH Olsen RH Mounier R. A 2-wk reduction of ambulatory activity attenuates peripheral insulin sensitivity. J Appl Physiol. (2010) 108:1034–40. 10.1152/japplphysiol.00977.2009 20044474

[17] Hagobian TA Braun B. Interactions between energy surplus and short-term exercise on glucose and insulin responses in healthy people with induced, mild insulin insensitivity. Metabolism (2006) 55:402–8. 10.1016/j.metabol.2005.09.017 16483886

[18] Chaput JP Drapeau V Poirier P Teasdale N Tremblay A. Glycemic instability and spontaneous energy intake: association with knowledge-based work. Psychosom Med. (2008) 70:797–804. 10.1097/PSY.0b013e31818426fa 18725427

[19] Chaput JP Tremblay A. The glucostatic theory of appetite control and the risk of obesity and diabetes. Int J Obes. (2009) 33:46–53. 10.1038/ijo.2008.221 19002144

[20] Tremblay A Despres JP Leblanc C Craig CL Ferris B Stephens T. Effect of intensity of physical activity on body fatness and fat distribution. Am J Clin Nutr. (1990) 51:153–7. 10.1093/ajcn/51.2.153 2305702

[21] Yoshioka M Doucet E St-Pierre S Almeras N Richard D Labrie A. Impact of high-intensity exercise on energy expenditure, lipid oxidation and body fatness. Int J Obes Relat Metab Disord. (2001) 25:332–9. 10.1038/sj.ijo.0801554 11319629

[22] Tremblay A Simoneau JA Bouchard C. Impact of exercise intensity on body fatness and skeletal muscle metabolism. Metabolism (1994) 43:814–8. 10.1016/0026-0495(94)90259-3 8028502

[23] Imbeault P Saint-Pierre S Almeras N Tremblay A. Acute effects of exercise on energy intake and feeding behaviour. Br J Nutr. (1997) 77:511–21. 10.1079/BJN19970053 9155502

[24] Tremblay A Coveney S Despres JP Nadeau A Prud'homme D. Increased resting metabolic rate and lipid oxidation in exercise-trained individuals: evidence for a role of beta-adrenergic stimulation. Can J Physiol Pharmacol. (1992) 70:1342–7. 10.1139/y92-188 1337012

[25] Ewbank PP Darga LL Lucas CP. Physical activity as a predictor of weight maintenance in previously obese subjects. Obes Res. (1995) 3:257–63. 10.1002/j.1550-8528.1995.tb00146.x 7627774

[26] Chaput JP Lambert M Mathieu ME Tremblay MS O' Loughlin J Tremblay A. Physical activity vs. sedentary time: independent associations with adiposity in children. Pediatr Obes. (2012) 7:251–8. 10.1111/j.2047-6310.2011.00028.x 22461356

[27] Healy GN Matthews CE Dunstan DW Winkler EA Owen N. Sedentary time and cardio-metabolic biomarkers in US adults: NHANES 2003-06. Eur Heart J. (2011) 32:590–7. 10.1093/eurheartj/ehq451 21224291 PMC3634159

[28] Saunders TJ Tremblay MS Mathieu ME Henderson M O'Loughlin J Tremblay A. Associations of sedentary behavior, sedentary bouts and breaks in sedentary time with cardiometabolic risk in children with a family history of obesity. PLoS ONE (2013) 8:e79143. 10.1371/journal.pone.0079143 24278117 PMC3835898

[29] Henderson M Gray-Donald K Mathieu ME Barnett TA Hanley JA O'Loughlin J. How are physical activity, fitness, and sedentary behavior associated with insulin sensitivity in children? Diabetes Care (2012) 35:1272–8. 10.2337/dc11-1785 22492585 PMC3357250

[30] Ekelund U Steene-Johannessen J Brown WJ Fagerland MW Owen N Powell KE. Does physical activity attenuate, or even eliminate, the detrimental association of sitting time with mortality? A harmonised meta-analysis of data from more than 1 million men and women. Lancet (2016) 388:1302–10. 10.1016/S0140-6736(16)30370-1 27475271

[31] Hobbs M Pearson N Foster PJ Biddle SJ. Sedentary behaviour and diet across the lifespan: an updated systematic review. Br J Sports Med. (2015) 49:1179–88. 10.1136/bjsports-2014-093754 25351783

[32] Mitter S. Globalization, technological changes and the search for a new paradigm for women's work. Gend Technol Dev. (1999) 3:3–17. 12179936

[33] Chaput JP Tremblay A. Obesity and physical inactivity: the relevance of reconsidering the notion of sedentariness. Obes Facts (2009) 2:249–54. 10.1159/000227287 20054231 PMC6515935

[34] McCann BS Warnick GR Knopp RH. Changes in plasma lipids and dietary intake accompanying shifts in perceived workload and stress. Psychosom Med. (1990) 52:97–108. 10.1097/00006842-199001000-00008 2305026

[35] Benedict FG Benedict CG. The energy requirements of intense mental effort. Proc Natl Acad Sci USA. (1930) 16:438–43. 10.1073/pnas.16.6.438 16587597 PMC526666

[36] Chaput JP Tremblay A. Acute effects of knowledge-based work on feeding behavior and energy intake. Physiol Behav. (2007) 90:66–72. 10.1016/j.physbeh.2006.08.030 17023010

[37] Epel E Lapidus R McEwen B Brownell K. Stress may add bite to appetite in women: a laboratory study of stress-induced cortisol and eating behavior. Psychoneuroendocrinology (2001) 26:37–49. 10.1016/S0306-4530(00)00035-4 11070333

[38] Gluck ME. Stress response and binge eating disorder. Appetite (2006) 46:26–30. 10.1016/j.appet.2005.05.004 16260065

[39] Michaud I Chaput JP O'Loughlin J Tremblay A Mathieu ME. Long duration of stressful homework as a potential obesogenic factor in children: a QUALITY study. Obesity (2015) 23:815–22. 10.1002/oby.21026 25755164

[40] Vander AJ Sherman JH Luciano DS Physiologie Humaine. Montreal: Chenelière/McGraw-Hill (1995).

[41] Volkow ND Fowler JS Wang GJ Telang F Logan J Wong C. Methylphenidate decreased the amount of glucose needed by the brain to perform a cognitive task. PLoS ONE (2008) 3:e2017. 10.1371/journal.pone.0002017 18414677 PMC2291196

[42] Myers A Gibbons C Finlayson G Blundell J. Associations among sedentary and active behaviours, body fat and appetite dysregulation: investigating the myth of physical inactivity and obesity. Br J Sports Med. (2017) 51:1540–4. 10.1136/bjsports-2015-095640 27044438

[43] Stubbs RJ Hughes DA Johnstone AM Horgan GW King N Blundell JE. A decrease in physical activity affects appetite, energy, and nutrient balance in lean men feeding ad libitum. Am J Clin Nutr. (2004) 79:62–9. 10.1093/ajcn/79.1.62 14684398

[44] Stephens BR Granados K Zderic TW Hamilton MT Braun B. Effects of 1 day of inactivity on insulin action in healthy men and women: interaction with energy intake. Metabolism (2011) 60:941–9. 10.1016/j.metabol.2010.08.014 21067784

[45] Granados K Stephens BR Malin SK Zderic TW Hamilton MT Braun B. Appetite regulation in response to sitting and energy imbalance. Appl Physiol Nutr Metab. (2012) 37:323–33. 10.1139/h2012-002 22462636

[46] Murgatroyd PR Goldberg GR Leahy FE Gilsenan MB Prentice AM. Effects of inactivity and diet composition on human energy balance. Int J Obes Relat Metab Disord. (1999) 23:1269–75. 10.1038/sj.ijo.0801062 10643683

[47] Chaput JP Despres JP Bouchard C Tremblay A. Association of sleep duration with type 2 diabetes and impaired glucose tolerance. Diabetologia (2007) 50:2298–304. 10.1007/s00125-007-0786-x 17717644

[48] Chaput JP Després JP Bouchard C Tremblay A. The association between sleep duration and weight gain in adults: a 6-year prospective study from the quebec family study. Sleep (2008) 31:517–23. 10.1093/sleep/31.4.517 18457239 PMC2279744

[49] World Health Organization Global Recommendations on Physical Activity for Health. Geneva (2010). 26180873

[50] Lemay V Drapeau V Tremblay A Mathieu ME. Exercise and negative energy balance in males who perform mental work. Pediatr Obes. (2014) 9:300–9. 10.1111/j.2047-6310.2013.00158.x 23629946

[51] Neumeier WH Goodner E Biasini F Dhurandhar EJ Menear KS Turan B. Exercise following mental work prevented overeating. Med Sci Sports Exerc. (2016) 48:1803–9. 10.1249/MSS.0000000000000961 27116647 PMC4987226

[52] Tremblay A Lachance E. Tackling obesity at the community level by integrating healthy diet, movement and non-movement behaviours. Obes Rev. (2017) 18(Suppl. 1):82–7. 10.1111/obr.12504 28164447

[53] Lapointe T Brassard P Rattray B Perusse-Lachance E. Physical activity counteracts the influence of mental work on blood pressure in healthy children. Physiol Behav. (2016) 164(Pt A):102–6. 10.1016/j.physbeh.2016.05.048 27241633

[54] Lanningham-Foster L Foster RC McCrady SK Manohar CU Jensen TB Mitre NG. Changing the school environment to increase physical activity in children. Obesity (2008) 16:1849–53. 10.1038/oby.2008.282 18535550 PMC2690697

[55] Tremblay A Mathieu ME Chaput JP. A sound mind in a sound body. Obesity (2009) 17:631. 10.1038/oby.2008.546 19322151

[56] Rempel D Krause N Do sit-stand workstations improve cardiovascular health? J Occup Environ Med. (2018) 60:e319–20. 10.1097/JOM.0000000000001351 29677023

[57] MacEwen BT Saunders TJ MacDonald DJ Burr JF. Sit-stand desks to reduce workplace sitting time in office workers with abdominal obesity: a randomized controlled trial. J Phys Act Health (2017) 14:710–5. 10.1123/jpah.2016-0384 28513245

[58] Dutta N Koepp GA Stovitz SD Levine JA Pereira MA. Using sit-stand workstations to decrease sedentary time in office workers: a randomized crossover trial. Int J Environ Res Public Health (2014) 11:6653–65. 10.3390/ijerph110706653 24968210 PMC4113835

[59] Jerome M Janz KF Baquero B Carr LJ. Introducing sit-stand desks increases classroom standing time among university students. Prev Med Rep. (2017) 8: 232–7. 10.1016/j.pmedr.2017.10.019 29159019 PMC5683670

